# Synthesis, characterization, biological evaluation and molecular docking studies of 2-(1H-benzo[d]imidazol-2-ylthio)-*N*-(substituted 4-oxothiazolidin-3-yl) acetamides

**DOI:** 10.1186/s13065-017-0361-6

**Published:** 2017-12-22

**Authors:** Snehlata Yadav, Balasubramanian Narasimhan, Siong M. Lim, Kalavathy Ramasamy, Mani Vasudevan, Syed Adnan Ali Shah, Manikandan Selvaraj

**Affiliations:** 1 Department of Pharmaceutical Sciences, Maharshi Dayanand University, Rohtak, 124001 India; 2 Faculty of Pharmaceutical Sciences, Maharshi Dayanand University, Rohtak, 124001 India; 3 Faculty of Pharmacy, Universiti Teknologi MARA (UiTM), 42300 Bandar Puncak Alam, Selangor Darul Ehsan Malaysia; 4 Collaborative Drug Discovery Research (CDDR) Group, Brain and Neuroscience Communities of Research, Universiti Teknologi MARA (UiTM), 40450 Shah Alam, Selangor Darul Ehsan Malaysia; 5 Department of Pharmacology and Toxicology, College of Pharmacy, Qassim University, Buraidah, 51452 Kingdom of Saudi Arabia; 6 Atta-ur-Rahman Institute for Natural Products Discovery (AuRIns), Universiti Teknologi MARA, Puncak Alam Campus, 42300 Bandar Puncak Alam, Selangor D. E. Malaysia; 7 Integrative Pharmacogenomics Institute (iPROMISE), Universiti Teknologi MARA (UiTM), Puncak Alam Campus, 42300 Bandar Puncak Alam, Selangor Darul Ehsan Malaysia

**Keywords:** Benzimidazole derivatives, Molecular modeling, Cytotoxic, Antimicrobial activity, CDK8

## Abstract

**Background:**

A series of 2-(1H-benzo[d]imidazol-2-ylthio)-*N*-(substituted 4-oxothiazolidin-3-yl) acetamides was synthesized and characterized by physicochemical and spectral means. The synthesized compounds were evaluated for their in vitro antimicrobial activity against *Staphylococcus aureus*, *Bacillus subtilis*, *Escherichia coli*, *Candida albican*s and *Aspergillus niger* by tube dilution method. The in vitro cytotoxicity study of the compounds was carried out against human colorectal (HCT116) cell line. The most promising anticancer derivatives (**5l, 5k, 5i** and **5p**) were further docked to study their binding efficacy to the active site of the cyclin-dependent kinase-8.

**Results:**

All the compounds possessed significant antimicrobial activity with MIC in the range of 0.007 and 0.061 µM/ml. The cytotoxicity study revealed that almost all the derivatives were potent in inhibiting the growth of HCT116 cell line in comparison to the standard drug 5-fluorouracil. Compounds **5l** and **5k** (IC_50_ = 0.00005 and 0.00012 µM/ml, respectively) were highly cytotoxic towards HCT116 cell line in comparison to 5-fluorouracil (IC_50_ = 0.00615 µM/ml) taken as standard drug.

**Conclusion:**

The molecular docking studies of potent anticancer compounds **5l, 5k, 5i** and **5p** showed their putative binding mode and significant interactions with cyclin-dependent kinase-8 as prospective agents for treating colon cancer.

## Background

The advancement in the field of science and technology has made incredible progress in the field of medicine leading to the discovery of many drugs. Antibiotics are one of the significant therapeutic discoveries of the 20th century in combating the battle against life-threatening microbial infections [1]. However, multi-drug resistant infections are of particular concern as it causes an annual toll of about 25,000 patients, even in the European countries [2]. Over the past few decades, the problems posed by multi-drug resistant microorganisms have reached an alarming level leading to a serious challenge to the medical community [3]. The conscious usage of the currently marketed antibiotics is the one way to fight with this challenge and the other being the development of newer antimicrobial agents with novel mechanism of action and enhanced activity profile [4, 5].

The word “cancer” includes a vast group of diseases affecting almost any body part and represents the speedy formation of unusual cells leading to malignancy on growing beyond their usual boundaries [6]. Colorectal cancer (CRC) is one of the most prevailing cancers in developed regions of the world. It is ranked third among common malignancies in the world after breast and lung cancers with an estimated global toll of 579,000 in year 2000. CRC may be associated with dietary factors or may be the result of accumulation of genetic changes throughout the life of person within the epithelial cells of the mucosal surface of the bowel wall or may be inherited from the family members which accounts for only 10% of it [7–9]. The modern treatment remedies mainly reckon on chemotherapeutics and monoclonal antibodies in addition to surgical intervention for the treatment of advanced and metastasized colon carcinoma. The targeted as well as combination therapy has perked up the outcomes for CRC patients. However, late diagnosis of the disease often accompanied by metastases and high recurrence rates seek major lethality problems [10, 11]. Despite leading upsurge in technology and scientific proficiency into drug research and development processes, drug resistance sustains as a prime justification in the pharmacotherapy of all cancers [12]. It is a hard to believe fact that during the last decade, nearly 50% drugs has been approved by the US Food and Drug Administration [13] and hence we are continuously facing a dearth of innovative medicinal agents to combat the battle against the monster. In pursuit of these goals, our research efforts are focused on the development of novel structural moieties with promising antimicrobial and anticancer properties.

Cyclin-dependent kinase-8 (CDK8) has been reported to regulate basal transcription by phosphorylation of RNA polymerase II8 and to phosphorylate E2F1, thereby activating Wnt signaling. CDK8 gene expression correlates with activation of β-catenin, a core transcriptional regulator of canonical Wnt signaling in colon and gastric cancers. Interestingly, CDK8 gene expression also correlates with increased mortality in colorectal, breast, and ovarian cancers [14].

Benzimidazole is a heterocyclic moiety of immense importance in drug discovery [15]. Moreover, the structural analogy of benzimidazole to the biological nucleotides enable it to interact with the biopolymers while enriching it with vast number of therapeutic activities including anticancer, antibacterial, antifungal, antiviral, anthelmintic, antihypertensive, antioxidant and anticoagulant activities [16].

Recent literature reveals that the thiazolidinone moiety is one of the most extensively studied heterocyclic moiety for its biological activities. The current drug design trend is to club two or three heterocyclic molecules having different sites of action to serve as a new scaffold towards the development of novel biologically active agents [17]. Thiazolidinones containing imidazole, benzimidazole, acridine, thiazole, quinazolin-4(3H)-one, *syn*-triazine, pyridine, or diazine fragments is a wonder nucleus that exhibits appreciable antibacterial, antimicrobial, antitumor, anti-HIV and anticancer activities [18–21].

In light of above facts and in continuation of our efforts in search of novel antimicrobial and anticancer agents, in the present study, we hereby report the synthesis, antimicrobial, anticancer and molecular docking studies of 2-(1H-benzo[d]imidazol-2-ylthio)-*N*-(substituted 4-oxothiazolidin-3-yl) acetamides [22, 23].

## Results and discussion

### Chemistry

**Scheme 1:**
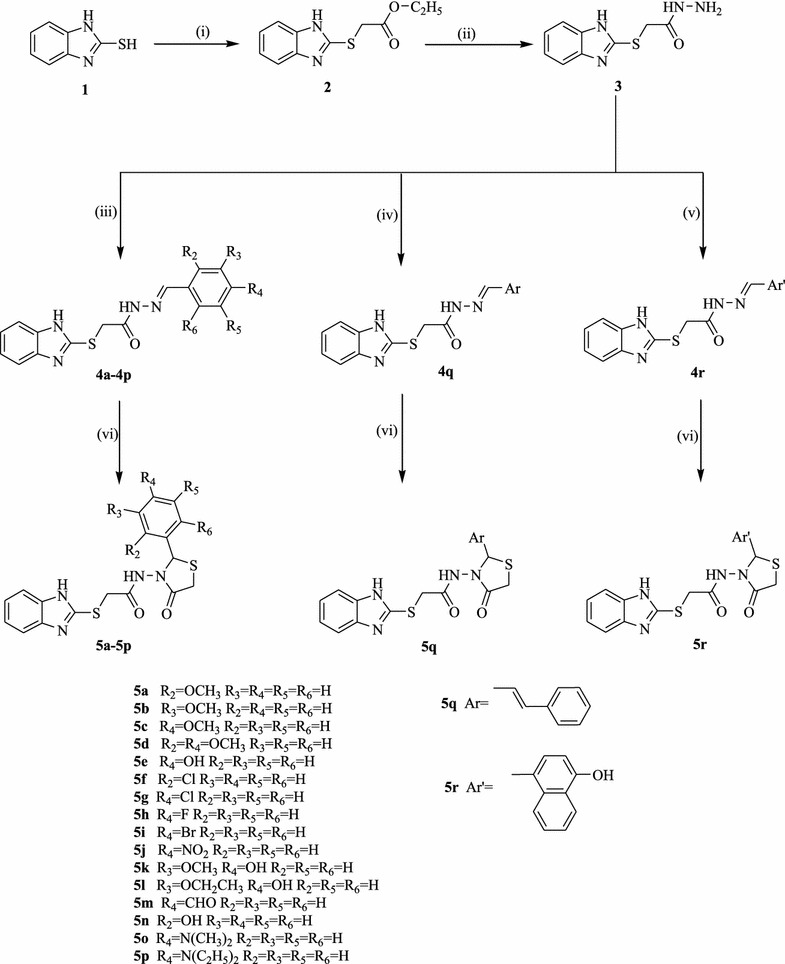
Scheme for synthesis of benzimidazole-substituted-1,3-thiazolidin-4-ones. Reaction conditions: (i) Ethanol, ethyl chloroacetate, stirring for 24 h. (ii) Ethanol, hydrazine hydrate, reflux. (iii) Aryl aldehyde, ethanol, a few drops of glacial acetic acid. (iv) Cinnamaldehyde, ethanol, a few drops of glacial acetic acid. (v) 4-Hydroxy-naphthaldehyde, ethanol, a few drops of glacial acetic acid. (vi) Dioxane, thioglycolic acid, anhydrous zinc chloride, reflux

A series of benzimidazole-substituted-1,3-thiazolidin-4-ones (**5a–5r**) was synthesized as depicted in Scheme 1. The structures assigned to the synthesized compounds **5a–5r** on the basis of IR, ^1^HNMR and ^13^CNMR spectroscopic data are in accordance with the proposed molecular structures. The formation of ester from 2-mercaptobenzimidazole is confirmed by absence S–H stretching at 2600–2550 cm^−1^ in the IR spectra. The appearance of C=O stretch in the range of 1680–1630 cm^−1^and N–H stretch 3100–3070 cm^−1^ indicated the formation of secondary amide (**5a–5r**) synthesized by the reaction of ester and hydrazine hydrate. Further, –OCN deformation at around 630–530 cm^−1^ also confirmed the formation of secondary amide. The presence of N–H stretching at 3500 cm^−1^ confirmed the formation of hydrazide derivative. The appearance of C–O–C stretch of aralkyl confirmed presence of methoxy group in compounds **5a, 5b, 5c** and **5k,** dimethoxy group in compound **5d** and ethoxy group in compound **5l**. The aryl nitro group in compound **5j** was assured by the appearance of C–N stretch in the range of 833 cm^−1^. Appearance of a wide broad peak in the range of 3200–2500 cm^−1^ accounted for presence of –OH group associated with C=O in compounds **5e**, **5l, 5n** and **5r**. The C–H stretch at 2832 cm^−1^for aldehyde group confirmed the aromatic aldehyde group in compound **5** **m**. The tertiary amine in compounds **5o** and **5p** was confirmed by C–N stretch at 1362 cm^−1^.

The multiplet corresponding to δ 6.8–7.9 ppm confirmed the presence of aromatic protons of aryl nucleus and benzimidazole. A singlet at around δ 3.30 ppm confirmed the methylene of thiazolidinone and the presence of hydrogen of secondary amide was confirmed by a singlet around δ 8.0 ppm. The presence of methoxy group in compounds **5a–5d** and **5k** was confirmed due to singlet at around δ 3.38 ppm. The doublet at δ 6.58 ppm with coupling constant of 12 Hz confirmed the presence of aliphatic double bond (C=C) in compound **5q**. In ^13^CNMR analysis of the synthesized compounds, singlets for carbons of CH_2_ and CH of thiazolidinone ring were obtained at around δ 35 and δ 40 ppm, respectively. The aromatic carbons appeared between δ 110–153 ppm. The appearance of peak at around δ 160 ppm confirmed the presence of carbon of amide. Further confirmation was made on the basis of mass analysis. The results of elemental (CHN) analysis are within acceptable limits (± 0.4%).

### In vitro antimicrobial activity

The results of antimicrobial activity (MIC and MBC/MFC values) of the synthesized benzimidazole derivatives and standard drugs using *Escherichia coli* MTCC 1652 (Gram-negative bacterial strain); *Bacillus subtilis* MTCC 2063, *Staphylococcus aureus* MTCC 2901 (Gram-positive bacterial strains); *Candida albicans* MTCC 227 and *Aspergillus niger* MTCC 8189 (fungal strains) are presented in Tables 1, 2 respectively. All the synthesized benzimidazole derivatives were potent antimicrobial agents in comparison to norfloxacin and fluconazole taken as standard antibacterial and antifungal drugs, respectively. Among the synthesized derivatives, compounds **5i (**MIC = 0.027 µM/ml**)** containing bromo and **5p (**MIC = 0.027 µM/ml**)** containing diethylamino substituent effectively inhibited the growth of *S. aureus* and *A. niger,* respectively. Compounds **5d** and **5g** having 2,4-dimethoxy and 4-chloro substituents respectively, were found to be best antibacterial agents against *B. subtilis* with MIC = 0.014 and 0.015 µM/ml, respectively. Compounds **5** **k (**MIC = 0.007 µM/ml) with 3-methoxy-4-hydroxy and **5n** (MIC = 0.008 µM/ml) with 4-hydroxy substitution potentially inhibited the growth of Gram negative bacterial strain, *E. coli.* Compounds **5g, 5i** and **5j** also inhibited the growth of *E. coli* but to a lesser extent than compounds **5k** and **5n.** Compound **5j** (MIC = 0.007 µM/ml) exhibited high efficacy against *C. albicans* as compared to fluconazole (MIC of 0.50 µM/ml).

**Table 1:** MIC of benzimidazole-substituted-1,3-thiazolidin-4-ones in µM/ml

Comp. no.	MIC in µM/ml				
	*S. aureus*	*B. subtilis*	*E. coli*	*C. albicans*	*A. niger*
**5a**	0.030	0.030	0.030	0.060	0.030
**5b**	0.060	0.030	0.030	0.030	0.030
**5c**	0.030	0.030	0.030	0.030	0.030
**5d**	0.028	0.014	0.028	0.028	0.028
**5e**	0.031	0.031	0.031	0.031	0.031
**5f**	0.030	0.030	0.030	0.030	0.030
**5g**	0.030	0.015	0.015	0.030	0.030
**5h**	0.031	0.031	0.031	0.031	0.031
**5i**	0.027	0.027	0.013	0.027	0.027
**5j**	0.029	0.029	0.015	0.007	0.029
**5k**	0.058	0.029	0.007	0.029	0.029
**5l**	0.028	0.028	0.028	0.028	0.028
**5m**	0.061	0.030	0.030	0.030	0.030
**5n**	0.031	0.031	0.008	0.031	0.031
**5o**	0.029	0.029	0.029	0.029	0.029
**5p**	0.027	0.027	0.027	0.027	0.027
**5q**	0.030	0.030	0.030	0.030	0.030
**5r**	0.028	0.028	0.028	0.028	0.028
Norfloxacin	0.47	0.47	0.47	–	–
Fluconazole	–	–	–	0.50	0.50

**Table 2:** MBC/MFC of benzimidazole-substituted-1,3-thiazolidin-4-ones in µM/ml

Comp. no.	MBC in µM/ml				
	*S. aureus*	*B. subtilis*	*E. coli*	*C. albicans*	*A. niger*
**5a**	> 0.121	> 0.121	> 0.121	0.060	0.060
**5b**	> 0.121	> 0.121	0.060	0.060	0.121
**5c**	> 0.121	> 0.121	0.030	0.060	0.030
**5d**	> 0.112	> 0.112	0.056	0.056	0.112
**5e**	> 0.125	> 0.125	0.062	0.062	0.062
**5f**	> 0.119	> 0.119	0.030	0.060	0.060
**5g**	> 0.119	0.119	0.015	0.060	0.119
**5h**	> 0.124	> 0.124	0.062	0.062	0.124
**5i**	> 0.108	> 0.108	0.013	0.054	0.054
**5j**	> 0.116	> 0.116	0.015	0.015	0.116
**5k**	> 0.116	> 0.116	0.058	0.058	0.116
**5l**	> 0.112	> 0.112	0.056	0.056	0.056
**5m**	> 0.121	> 0.121	0.061	0.061	0.121
**5n**	> 0.122	> 0.122	0.030	0.061	0.030
**5o**	> 0.125	> 0.125	0.031	0.062	0.125
**5p**	> 0.117	> 0.117	0.029	0.058	0.058
**5q**	> 0.110	0.110	0.055	0.055	0.110
**5r**	> 0.111	0.111	0.055	0.055	0.055

From the results of MBC/MFC (Table 2), it was concluded that none of the derivatives were bactericidal except for compounds **5i** and **5j** which were bactericidal against *E. coli*. However, compounds **5c** and **5j** were fungicidal against *A. niger* and *C. albicans,* respectively.

### In vitro cytotoxicity

Most of the synthesized derivatives inhibited the proliferation of HCT116 (human colorectal) cell line to a better extent as compared to 5-fluorouracil used as standard drug (Table 3). However, 3-ethoxy-4-hydroxy substituted compound, **5l** and 3-methoxy-4-hydroxy substituted compound, **5k** are the most potent ones with IC_50_ of 0.00005 and 0.00012 µM/ml respectively when compared to 5-fluorouracil (IC_50_ = 0.00615 µM/ml). Compounds **5b** and **5m** were the most inactive derivatives among the series.

**Table 3:** IC_50_ (in µM/ml) values for cytotoxicity screening of synthesized compounds on HCT116 cell lines

Comp. no.	IC_50_ (µM/ml)
**5a**	0.00869
**5b**	0.24125
**5c**	0.01351
**5d**	0.00099
**5e**	0.01748
**5f**	0.00477
**5g**	0.00716
**5h**	0.07454
**5i**	0.00065
**5j**	0.00256
**5k**	0.00012
**5l**	0.00005
**5m**	0.24243
**5n**	0.00731
**5o**	0.00999
**5p**	0.00094
**5q**	0.00176
**5r**	0.00888
5-Fluorouracil	0.00615

### Docking studies and binding mode analysis

**Fig. 1:**
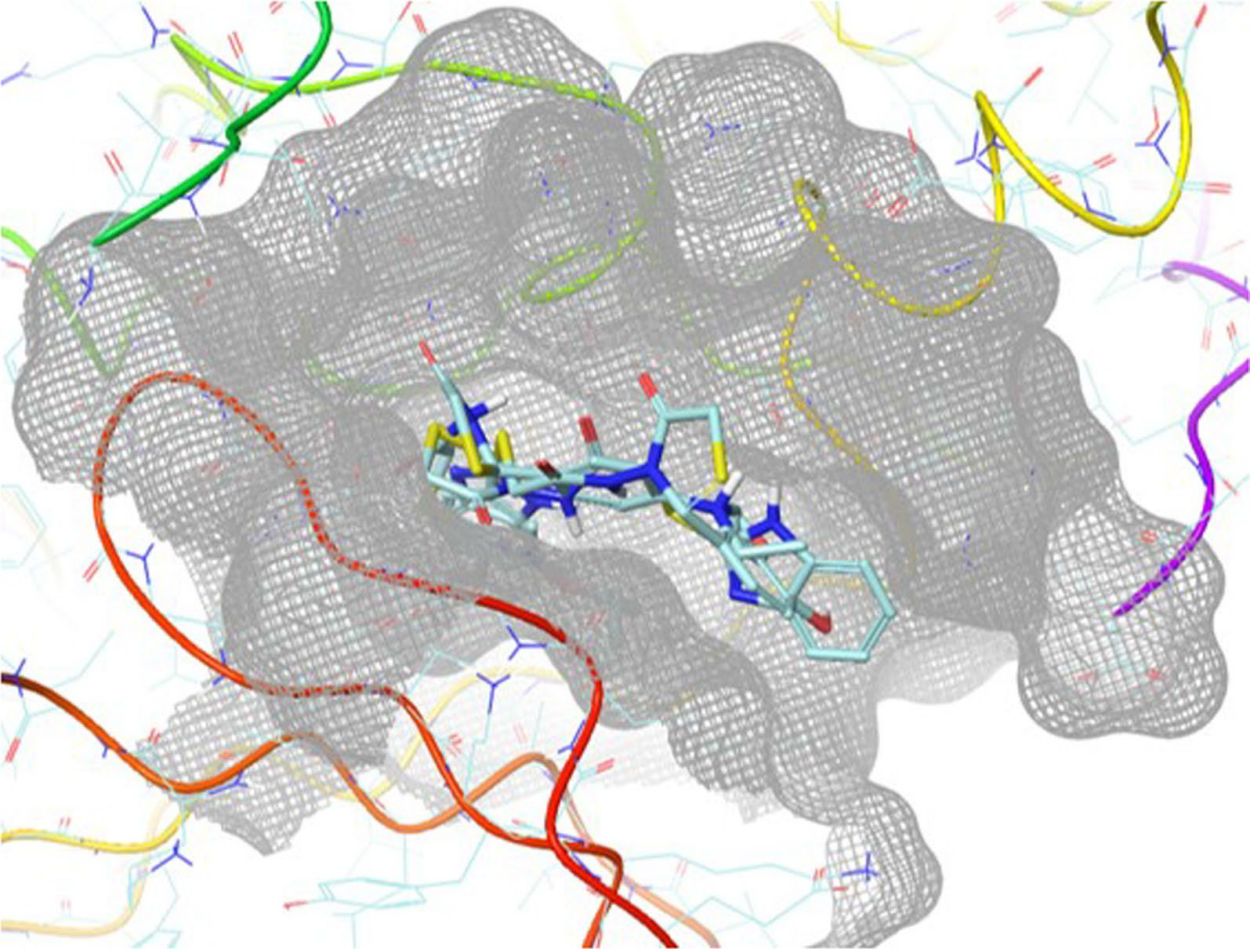
Binding mode of compounds **5l, 5k, 5i** and **5p** in CDK8 active site represented as surface

**Fig. 2:**
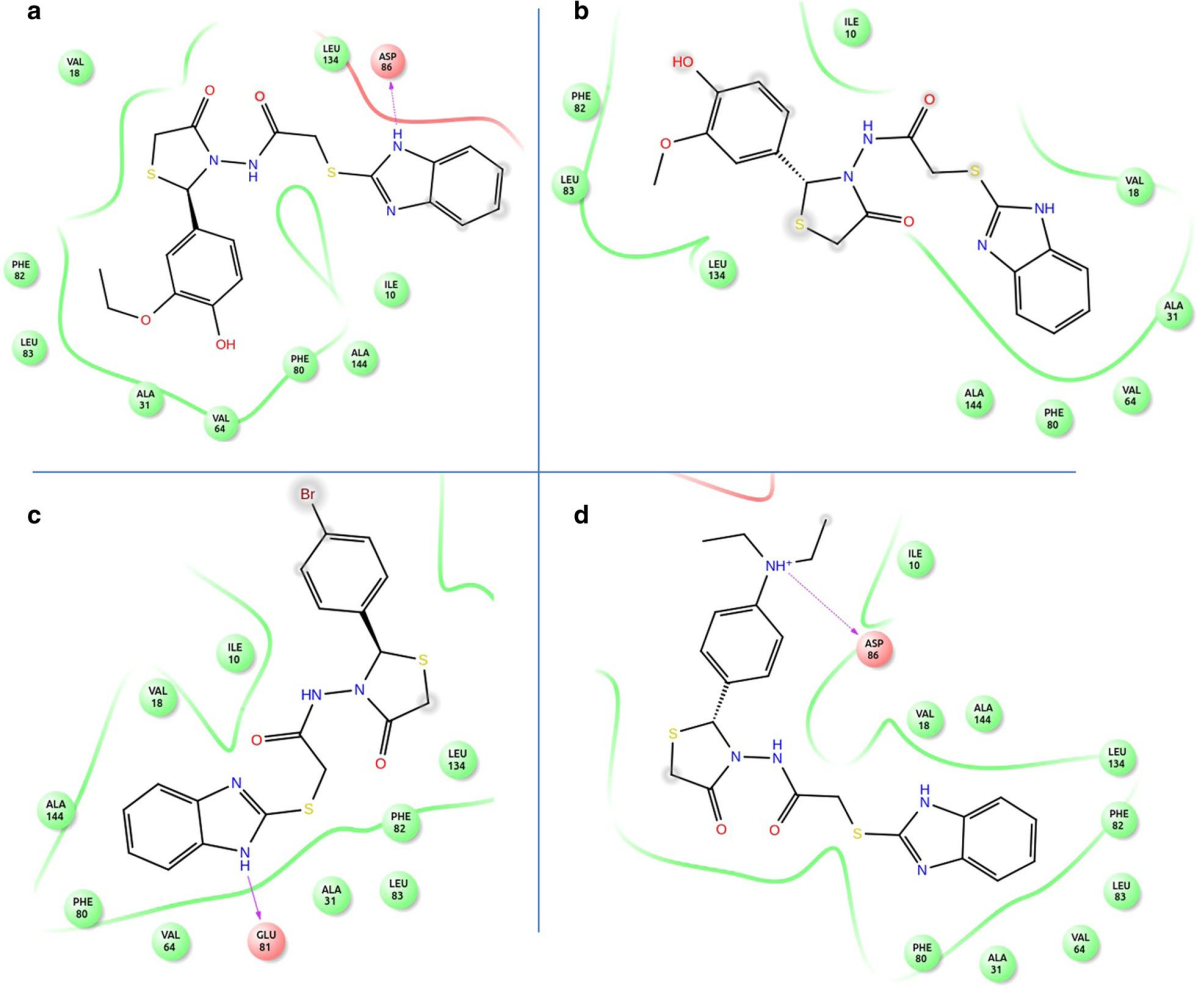
Graphical illustration of predicted binding mode in the active site of CDK8 for **a** compound **5l**, **b** compound **5k**, **c** compound **5i** and **d** compound **5p**. Key residues involved in the interactions are labelled and the compounds are represented as lines. The hydrogen bond interactions are represented by magenta arrow

Molecular modeling studies were accomplished using Glide docking tool. The possible binding mode of the synthesized derivatives was targeted on cyclin-dependent kinase (CDK8) crystal structure. The co-complexed 5XG ligand of 20 Å radius was used as reference and all the derivatives were docked into the active site of CDK8. The results were analyzed based on XP mode and XPG score scoring function. The docked binding mode was analyzed for interactions between compounds and the key residues of CDK8. Here, we have discussed in detail the binding modes of the four most active compounds *i.e.,* **5l, 5k, 5i** and **5p**. Figure 1 shows the binding mode of these most active compounds onto the active site of CDK8. Compound **5l** is positioned in the ravine of active site of CDK8 due to hydrogen bonding between the imidazole and Asp86. The complex of compound **5l** and amino acid residues of CDK8 such as Ile10, Val18, Ala31, Val64, Phe80, Phe82, Leu83, Leu134 and Ala144 is stabilized due to the presence of hydrophobic interaction between them (Fig. 2a).

The hydrophobic interaction of the imidazole ring of compound **5k** (Fig. 2b) with residues such as Val18, Ala31, Val64, Phe80 and Ala144 stabilizes the entire complex. The 2-ethoxyphenol ring of compound **5k** forms non-polar interactions with Ile10, Phe82, Leu83 and Leu134. In spite of bearing polar moieties, the orientation of this compound was in such a manner that it could not form hydrogen bond with key polar residues of the active site of CDK8.

The NH of imidazole ring in compound **5i** forms hydrogen bond with Glu81 residue of the enzyme while the rest of the complex is stabilized by hydrophobic interactions with Ile10, Val18, Ala31, Val64, Phe80, Phe82, Leu134 and Ala144 residues (Fig. 2c).

In case of compound **5p,** the NH of *N*, *N*-diethylanilinium forms hydrogen bond with Asp86 and stabilizes the complex by the hydrogen bonding. The key residues Ile10, Val18, Ala31, Val64, Phe80, Phe82, Leu83, Leu134 and Ala144 of CDK8 are involved in the non-polar interaction as shown in Fig. 2d. From the active inhibition interaction pattern of the above four compounds, we concluded that stabilization of most of the complex by the hydrophobic interactions and further by hydrogen bonding considerably contributes towards the activity profile of the compounds.

## Conclusion

This work is focused on development of novel antimicrobial and cytotoxic agents against human colorectal cancer cell line based on 2-(1H-benzo[d]imidazol-2-ylthio)-*N*-(substituted 4-oxothiazolidin-3-yl) acetamides. A total of eighteen derivatives were synthesized using 2-mercaptobenzimidazole as starting compound and were characterized by physicochemical and spectral means. The antimicrobial evaluation was performed against Gram positive bacterial strains (*B. subtilis* and *S. aureus*) and Gram negative bacterial strain (*E. coli*) and fungi (*A. niger* and *C. albicans*) by tube dilution method. All the synthesized derivatives exhibited MIC range between 0.007 and 0.061 µM/ml and inhibited the microbial growth of much efficiently as compared to norfloxacin and fluconazole. The results of in vitro cytotoxicity against HCT116 cell line illustrated that all the synthesized derivatives were highly cytotoxic in comparison to 5-fluorouracil used as standard drug. Compounds **5l** and **5k** (IC_50_ = 0.00005 and 0.00012 µM/ml respectively) were highly cytotoxic towards HCT116 cell line in comparison to 5-fluorouracil. The molecular docking studies showed putative binding mode of the derivatives and their significant interactions with cyclin-dependent kinase-8 as prospective agents against colon cancer. The degree of activity and docking studies displayed by the novel innovative structural combination of benzimidazole and thiazolidinone rings make these compounds as new active leads to provide a powerful encouragement for further research in this area.

## Experimental

### Materials and methods

Reagents and chemicals of analytical grade were purchased from commercial sources and used as such without further purification. The melting points were determined on Labtech melting point apparatus and are uncorrected. The progress of reaction was confirmed by TLC performed on silica gel-G plates and the spot was visualized in iodine chamber. Media for antimicrobial activity were obtained from Hi-media Laboratories. Microbial type cell cultures (MTCC) for antimicrobial activity were procured from IMTECH, Chandigarh. Infrared (IR) spectra of the synthesized derivatives were obtained on Bruker 12060280, Software: OPUS 7.2.139.1294 spectrophotometer using KBr disc method covering a range of 4000–400 cm^−1^. The proton nuclear magnetic resonance (^1^H NMR) spectra were traced in deuterated dimethyl sulphoxide on Bruker Avance III 600 NMR spectrometer at a frequency of 600 MHz downfield to tetramethylsilane standard. Chemical shifts of ^1^H NMR were recorded as δ (parts per million). The ^13^C NMR of the compounds was obtained at a frequency of 150 MHz on Bruker Avance II 150 NMR spectrometer. The LCMS data were recorded on Waters Q-TOF micromass (ESI–MS) while elemental analyses were carried out on a Microprocessor based Thermo Scientific (FLASH 2000) CHNS-O Organic Elemental Analyser.

### General procedure for synthesis of ethyl-2-(1H-benzo[d]imidazol-2-ylthio)acetate (**2**)

A solution containing equimolar (0.03 mol) mixture of 2-mercaptobenzimidazole (**1**) and potassium hydroxide was heated to 80–90 °C along with stirring in 60 ml ethanol for 15 min. Ethyl chloroacetate (0.03 mol) was then added in one portion that resulted in rise of temperature of 30–40 °C due to exothermic reaction. The reaction mixture was stirred for 24 h at 18–20 °C and poured into 100 g of ice. The mixture was further stirred for 30 min, maintaining the temperature at 0–10 °C. The white product obtained was collected by filtration, washed to render it free of chloride, dried and recrystallized with ethanol to obtain pure product.

### General procedure for synthesis of ethyl-2-(1H-benzo[d]imidazol-2-ylthio) acetohydrazide (**3**)

A mixture of compound **2** (0.01 mol), hydrazine hydrate (0.06 mol) and absolute ethanol was gently refluxed in a round bottom flask on a water bath for an appropriate time. The completion of reaction was checked by TLC. The obtained mixture was concentrated and kept overnight in refrigerator. The creamish white precipitate obtained was separated from the mother liquor, dried and recrystallized from boiling water in order to obtain the pure compound **3**.

### General procedure for synthesis of Schiff’s bases (**4a**–**4r**)

A solution containing equimolar quantities of different aromatic aldehydes (0.01 mol) and compound **3** (0.01 mol) was refluxed for a period of 3–5 h using a few drops of glacial acetic acid as catalyst in ethyl alcohol. The completion of reaction was confirmed by TLC. The excess of solvent was distilled off at low temperature in a rotary evaporator. The resulting solid was washed with dilute ethyl alcohol and recrystallized from rectified spirit.

### General procedure for synthesis of benzimidazole-substituted-1,3-thiazolidin-4-ones (**5a**–**5r**)

The title compounds benzimidazole-substituted-1,3-thiazolidin-4-ones (**5a–5r**) were synthesised by refluxing the appropriate Schiff base (**4a–4r,** 0.015 M) with thioglycolic acid (0.015 M) for 8–10 h in 50 ml dioxane using a pinch of anhydrous zinc chloride as catalyst. The end-point of reaction was ascertained by TLC. The reaction mixture was then cooled to ambient temperature and neutralized with aqueous solution of sodium bicarbonate. The solid obtained was filtered, washed with water and recrystallized from ethanol.

### Spectral data of benzimidazole-substituted-1,3-thiazolidin-4-ones (**5a**–**5r**)

#### 2-(1H-Benzo[d]imidazol-2-ylthio)-*N*-(2-(2-methoxyphenyl)-4-oxothiazolidin-3-yl)acetamide (**5a**)

Yield 90.3%; mp 130–131 °C; R_f_ 0.46 (Toluene:Ethyl acetate: 3:1); IR (KBr cm^−1^) ν_max_: 574 OCN deformation, amide present, 1529 ring str. of thiazolidinone, 1595 C=O of thiazolidinone, 3071 N–H str. of imidazole; ^1^HNMR (DMSO-d_6_) δ: 3.33 (s, 2H of methylene), 7.01–7.98 (m, 8H aromatic), 6.99 (s, CH of thiazolidinone), 8.13 (s, NH of amide); ^13^C-NMR (DMSO-d_6_) δ: 35.74 CH_2_ of thiazolidinone, 40.01 CH_2_ aliphatic, 55.61 CH of thiazolidinone, 55.77 C of OCH_3_ (111.63, 111.98, 120.45, 121.03, 122.15, 130.99, 138.60,152.87, 157.19) C aromatic, 158.71 C=O of thiazolidinone, 162.25 C of amide; ESI–MS (m/z) [M + 1] ^+^ 415.51; Anal. Calcd. for C_19_H_18_N_4_O_3_S_2_: C, 55.05; H, 4.38; N, 13.52; O, 11.58; S, 15.47. Found: C, 55.02; H, 4.42; N, 13.56; O, 11.60; S, 15.50.

#### 2-(1H-Benzo[d]imidazol-2-ylthio)-*N*-(2-(3-methoxyphenyl)-4-oxothiazolidin-3-yl)acetamide (**5b**)

Yield 60.5%; mp 198–200 °C; R_f_ 0.34 (Toluene:Ethyl acetate: 3:1); IR (KBr cm^−1^) ν_max_: 533 OCN deformation, amide present, 1268 C–O–C of asymmetric aralkyl, 1466 ring str. of thiazolidinone, 1593 C=O of thiazolidinone, 2931 N–H str. of imidazole; ^1^HNMR (DMSO-d_6_) δ: 3.32 (s, 2H of methylene), 6.94–7.99 (m, 8H aromatic), 6.91 (s, CH of thiazolidinone), 8.00 (s, NH of amide); ^13^C-NMR (DMSO-d_6_) δ: 35.75 CH_2_ of thiazolidinone, 40.01 CH_2_ aliphatic, 162.28 C of amide; ESI–MS (m/z) [M + 1]^+^ 415.52; Anal. Calcd. for C_19_H_18_N_4_O_3_S_2_: C, 55.05; H, 4.38; N, 13.52; O, 11.58; S, 15.47. Found: C, 55.06; H, 4.41; N, 13.54; O, 11.59; S, 15.55.

#### 2-(1H-Benzo[d]imidazol-2-ylthio)-*N*-(2-(4-methoxyphenyl)-4-oxothiazolidin-3-yl)acetamide (**5c**)

Yield 81.2%; mp 205–208 °C; R_f_ 0.46 (Toluene:Ethyl acetate: 3:1); IR (KBr cm^−1^) ν_max_: 743 OCN deformation of amide, 1252 C–O–C str. aralkyl asymmetric, 1466 ring str. of thiazolidinone, 1634 C=O of thiazolidinone, 2931 N–H str. of imidazole, 3056 N–H str. secondary amide associated; ^1^HNMR (DMSO-d_6_) δ: 3.323 (s, 2H of methylene), 6.80–7.95 (m, 8H aromatic), 6.65 (s, CH of thiazolidinone), 7.98 (s, NH of amide); ^13^C-NMR (DMSO-d_6_) δ: 35.74 CH_2_ of thiazolidinone, 39.91 CH_2_ aliphatic, 55.05 CH of thiazolidinone, 55.21 C of OCH_3_ (114.06, 128.04) C aromatic, 160.04 C=O of thiazolidinone, 162.26 C of amide; ESI–MS (m/z) [M + 1]^+^ 415.52; Anal. Calcd. for C_19_H_18_N_4_O_3_S_2_: C, 55.05; H, 4.38; N, 13.52; O, 11.58; S, 15.47. Found: C, 55.03; H, 4.36; N, 13.52; O, 11.56; S, 15.43.

#### 2-(1H-Benzo[d]imidazol-2-ylthio)-*N*-(2-(2,4-dimethoxyphenyl)-4-oxothiazolidin-3-yl) acetamide (**5d**)

Yield 94.1%; mp 182–184 °C; R_f_ 0.31 (Toluene:Ethyl acetate: 3:1); IR (KBr cm^−1^) ν_max_: 743 OCN deformation of amide, 1034 C–O–C str. symmetric, 1463 ring str. of thiazolidinone, 1636 C=O of thiazolidinone, 2936 N–H str. of imidazole, 3057 N–H str. secondary amide; ^1^HNMR (DMSO-d_6_) δ: 3.33 (s, 2H of methylene), 6.96–7.95 (m, 8H aromatic), 6.69 (s, CH of thiazolidinone), 8.03 (s, NH of amide); ^13^C-NMR (DMSO-d_6_) δ: 35.73 CH_2_ of thiazolidinone, 39.89 CH_2_ aliphatic, 55.36 CH of thiazolidinone, 55.65 C of OCH_3_ (97.99, 106.21, 115.32, 126.80, 158.45) C aromatic, 161.85 C=O of thiazolidinone, 162.26 C of amide; ESI–MS (m/z) [M + 1]^+^ 445.24; Anal. Calcd. for C_20_H_20_N_4_O_4_S_2_: C, 55.04; H, 4.53; N, 12.60; O, 14.40; S, 14.43. Found: C, 55.07; H, 4.51; N, 12.57; O, 14.37; S, 14.45.

#### 2-(1H-Benzo[d]imidazol-2-ylthio)-*N*-(2-(4-hydroxyphenyl)-4-oxothiazolidin-3-yl)acetamide (**5e**)

Yield 73.2%; mp 105–107 °C; R_f_ 0.48 (Toluene:Ethyl acetate: 3:1); IR (KBr cm^−1^) ν_max_: 529 OCN deformation, amide present, 1508 ring str. of thiazolidinone, 1658 C=O of thiazolidinone, 2927 O–H associated conjugate chelation intramolecular H-bonded with C=O, 3060 N–H str. of secondary amide (associated), 3224 N–H str. of imidazole; ^1^HNMR (DMSO-d_6_) δ: 3.33 (s, 2H of methylene), 6.91–7.95 (m, 8H aromatic), 6.86 (s, CH of thiazolidinone), 8.55 (s, NH of amide); ^13^C-NMR (DMSO-d_6_) δ: 35.74 CH_2_ of thiazolidinone, 39.88 CH_2_ aliphatic, 40.00 CH of thiazolidinone, (115.05, 115.71, 127.52, 130.05) C aromatic, 162.27 C of amide; ESI–MS (m/z) [M + 1]^+^ 401.34; Anal. Calcd. for C_18_H_16_N_4_O_3_S_2_: C, 53.98; H, 4.03; N, 13.99; O, 11.99; S, 16.01. Found: C, 53.96; H, 3.98; N, 13.95; O, 11.96; S, 16.04.

#### 2-(1H-Benzo[d]imidazol-2-ylthio)-*N*-(2-(2-chlorophenyl)-4-oxothiazolidin-3-yl)acetamide (**5f**)

Yield 62.2%; mp 168–170 °C; R_f_ 0.42 (Toluene:Ethyl acetate: 3:1); IR (KBr cm^−1^) ν_max_: 755 C–Cl str. aromatic, 1498 ring str. of thiazolidinone, 1635 C=O of thiazolidinone, 3059 N–H str. of secondary amide (associated), 3206 N–H str. of imidazole; ^1^HNMR (DMSO-d_6_) δ: 3.33 (s, 2H of methylene), 7.00–7.95 (m, 8H aromatic), 8.16 (s, NH of amide); ^13^C-NMR (DMSO-d_6_) δ: 35.74 CH_2_ of thiazolidinone, 40.02 CH_2_ aliphatic, 41.14 CH of thiazolidinone, (126.45, 127.03, 127.12, 127.70, 128.16, 129.39, 129.48,130.14, 134.81, 158.21) C aromatic, 162.25 C=O of thiazolidinone, 167.52 C of amide; ESI–MS (m/z) [M + 1]^+^ 419.04; Anal. Calcd. for C_18_H_15_ClN_4_O_2_S_2_: C, 51.61; H, 3.61; N, 13.37; O, 7.64; S, 15.31. Found: C, 51.56; H, 3.59; N, 13.39; O, 7.67; S, 15.34.

#### 2-(1H-Benzo[d]imidazol-2-ylthio)-*N*-(2-(4-chlorophenyl)-4-oxothiazolidin-3-yl)acetamide (**5g**)

Yield 84.7%; mp 234–236 °C; R_f_ 0.37 (Toluene: Ethyl acetate:: 3:1); IR (KBr cm^−1^) ν_max_: 742 C–Cl str. aromatic, 1490 ring str. of thiazolidinone, 1636 C=O of thiazolidinone, 3059 N–H str. of secondary amide (associated), 3209 N–H str. of imidazole; ^1^HNMR (DMSO-d_6_) δ: 3.33 (s, 2H of methylene), 6.99-7.95 (m, 8H aromatic), 8.03 (s, NH of amide); ^13^C-NMR (DMSO-d_6_) δ: 35.74 CH_2_ of thiazolidinone, 39.76 CH_2_ aliphatic, 39.89 CH of thiazolidinone, (99.47, 112.61, 120.66, 128.23, 128.59, 133.46, 133.67,140.91) C aromatic, 162.25 C of amide; ESI–MS (m/z) [M + 1]^+^ 419.01; Anal. Calcd. for C_18_H_15_ClN_4_O_2_S_2_: C, 51.61; H, 3.61; N, 13.37; O, 7.64; S, 15.31. Found: C, 51.54; H, 3.65; N, 13.41; O, 7.65; S, 15.29.

#### 2-(1H-Benzo[d]imidazol-2-ylthio)-*N*-(2-(4-fluorophenyl)-4-oxothiazolidin-3-yl) acetamide (**5h**)

Yield 82.6%; mp 218-220 °C; R_f_ 0.43 (Toluene:Ethyl acetate: 3:1); IR (KBr cm^−1^) ν_max_: 744 OCN deformation, 1074 C–F str. monoflourinated compound, 1531 ring str. of thiazolidinone, 1632 C=O of thiazolidinone, 3058 N–H str. of secondary amide (associated), 3206 N–H str. of imidazole; ^1^HNMR (DMSO-d_6_) δ: 3.33 (s, 2H of methylene), 6.8–7.95 (m, 8H aromatic), 6.61 (s, CH of thiazolidinone), 8.00 (s, NH of amide); ^13^C-NMR (DMSO-d_6_) δ: 35.74 CH_2_ of thiazolidinone, 39.88 CH_2_ aliphatic, 40.00 CH of thiazolidinone, (112.10, 144.26) C aromatic, 162.26 C of amide; ESI–MS (m/z) [M + 1]^+^ 403.43; Anal. Calcd. for C_18_H_15_FN_4_O_2_S_2_: C, 53.72; H, 3.76; N, 13.92; O, 7.95; S, 15.93. Found: C, 53.74; H, 3.76; N, 13.95; O, 7.97; S, 15.91.

#### 2-(1H-Benzo[d]imidazol-2-ylthio)-*N*-(2-(4-bromophenyl)-4-oxothiazolidin-3-yl)acetamide (**5i**)

Yield 86.9%; mp 140–143 °C; R_f_ 0.38 (Toluene:Ethyl acetate: 3:1); IR (KBr cm^−1^) ν_max_: 626 OCN deformation, 744 C–Br str. aromatic, 1469 ring str. of thiazolidinone, 1595 C=O of thiazolidinone, 2815 N–H str. of imidazole; ^1^HNMR (DMSO-d_6_) δ: 3.34 (s, 2H of methylene), 7.01–7.95 (m, 8H aromatic), 8.02 (s, NH of amide); ^13^C-NMR (DMSO-d_6_) δ: 35.75 CH_2_ of thiazolidinone, 39.89 CH_2_ aliphatic, 40.02 CH of thiazolidinone, (122.32, 128.56, 130.16, 131.50, 131.97, 130.99, 132.88,133.92) C aromatic, 160.68 C=O of thiazolidinone, 162.26 C of amide; ESI–MS (m/z) [M + 1]^+^ 464.35; Anal. Calcd. for C_18_H_15_BrN_4_O_2_S_2_: C, 46.66; H, 3.26; N, 12.09; O, 6.91; S, 13.84. Found: C, 46.64; H, 3.23; N, 12.05; O, 6.95; S, 15.81.

#### 2-(1H-benzo[d]imidazol-2-ylthio)-*N*-(2-(4-nitrophenyl)-4-oxothiazolidin-3-yl)acetamide (**5j**)

Yield 88.8%; mp 120–122 °C; R_f_ 0.46 (Toluene:Ethyl acetate: 3:1); IR (KBr cm^−1^) ν_max_: 743 OCN deformation, 833 C–N str. aromatic nitro group, 1516 ring str. of thiazolidinone, 1597 C=O of thiazolidinone, 3211 N–H str. of imidazole; ^1^HNMR (DMSO-d_6_) δ: 3.35 (s, 2H of methylene), 6.50 (s, CH of thiazolidinone), 6.59–7.95 (m, 8H aromatic), 8.07 (s, NH of amide); ^13^C-NMR (DMSO-d_6_) δ: 35.73 CH_2_ of thiazolidinone, 39.75 CH_2_ aliphatic, 39.89 CH of thiazolidinone, (113.43, 113.79, 122.21, 123.80, 127.16, 128.53, 129.40, 150.71) C aromatic, 162.25 C of amide; ESI–MS (m/z) [M + 1]^+^ 430.43; Anal. Calcd. for C_18_H_15_N_5_O_4_S_2_: C, 50.34; H, 3.52; N, 16.31; O, 14.90; S, 14.93. Found: C, 50.29; H, 3.53; N, 16.35; O, 14.95; S, 14.91.

#### 2-(1H-Benzo[d]imidazol-2-ylthio)-*N*-(2-(4-hydroxy-3-methoxyphenyl)-4-oxothiazolidin-3-yl) acetamide (**5k**)

Yield 67.9%; mp 122–124 °C; R_f_ 0.76 (Toluene:Ethyl acetate: 3:1); IR (KBr cm^−1^) ν_max_: 616 OCN deformation, amide present, 1280 C–O–C str. of aralkyl asymmetric, 1465 ring str. of thiazolidinone, 1597 C=O of thiazolidinone, 3206 N–H str. of imidazole; ^1^HNMR (DMSO-d_6_) δ: 3.34 (s, 2H of methylene), 6.86–7.98 (m, 8H aromatic), 6.80 (s, CH of thiazolidinone), 8.57 (s, NH of amide); ^13^C-NMR (DMSO-d_6_) δ: 35.74 CH_2_ of thiazolidinone, 40.01 CH_2_ aliphatic, 55.48 CH of thiazolidinone, 55.83 C of OCH_3_ (99.47, 109.43, 115.32, 115.44, 121.37, 122.25, 147.93) C aromatic, 162.26 C of amide; ESI–MS (m/z) [M + 1]^+^ 431.47; Anal. Calcd. for C_19_H_18_N_4_O_4_S_2_: C, 53.01; H, 4.21; N, 13.01; O, 14.87; S, 14.90. Found: C, 52.97; H, 4.23; N, 13.05; O, 14.85; S, 14.94.

#### 2-(1H-Benzo[d]imidazol-2-ylthio)-*N*-(2-(3-ethoxy-4-hydroxyphenyl)-4-oxothiazolidin-3-yl) acetamide (**5l**)

Yield 89.9%; mp 110–112 °C; R_f_ 0.32 (Toluene:Ethyl acetate: 3:1); IR (KBr cm^−1^) ν_max_: 617 OCN deformation, amide present, 1276 C–O–C str. of aralkyl asymmetric, 1469 ring str. of thiazolidinone, 1637 C=O of thiazolidinone, 3063 N–H str. of secondary amide (associated), 3220 N–H str. of imidazole; ^1^HNMR (DMSO-d_6_) δ: 3.33 (s, 2H of methylene), 6.81–7.94 (m, 8H aromatic), 6.66 (s, CH of thiazolidinone), 7.95 (s, NH of amide); ^13^C-NMR (DMSO-d_6_) δ: 14.71 C of OCH_2_ CH_3_, 35.73 CH_2_ of thiazolidinone, 39.75 CH_2_ aliphatic, 39.88 CH of thiazolidinone, 64.03 C of OCH_2_CH_3_ (111.20, 115.49, 121.33, 125.86, 147.09, 148.74, 153.45) C aromatic, 162.26 C of amide; ESI–MS (m/z) [M + 1]^+^ 445.52; Anal. Calcd. for C_20_H_20_N_4_O_4_S_2_: C, 54.04; H, 4.53; N, 12.60; O, 14.40; S, 14.43. Found: C, 54.07; H, 4.55; N, 12.65; O, 14.43; S, 14.46.

#### 2-(1H-Benzo[d]imidazol-2-ylthio)-*N*-(2-(4-formylphenyl)-4-oxothiazolidin-3-yl)acetamide (**5m**)

Yield 69.2%; mp 200–203 °C; R_f_ 0.31 (Toluene:Ethyl acetate: 3:1); IR (KBr cm^−1^) ν_max_: 742 OCN deformation, amide present, 952 C-H out of plane bending of aldehyde group, 1468 ring str. of thiazolidinone, 1660 C=O of thiazolidinone, 3052 N–H str. of secondary amide (associated), 3192 N–H str. of imidazole; ^1^HNMR (DMSO-d_6_) δ: 3.33 (s, 2H of methylene), 6.95–7.85 (m, 8H aromatic), 6.91 (s, CH of thiazolidinone), 7.95 (s, NH of amide); ^13^C-NMR (DMSO-d_6_) δ: 35.75 CH_2_ of thiazolidinone, 40.01 CH_2_ aliphatic, 39.61 CH of thiazolidinone, 162.27 C of amide; ESI–MS (m/z) [M + 1]^+^ 413.44; Anal. Calcd. for C_19_H_16_N_4_O_3_S_2_: C, 55.32; H, 3.91; N, 13.58; O, 11.64; S, 15.55. Found: C, 55.37; H, 3.95; N, 13.55; O, 11.66; S, 15.58.

#### 2-(1H-Benzo[d]imidazol-2-ylthio)-*N*-(2-(2-hydroxyphenyl)-4-oxothiazolidin-3-yl)acetamide (**5n**)

Yield 62.4%; mp 196–198 °C; R_f_ 0.66 (Toluene:Ethyl acetate: 3:1); IR (KBr cm^−1^) ν_max_: 751 OCN deformation, amide present, 1466 ring str. of thiazolidinone, 1611 C=O of thiazolidinone, 2928 O–H associated with C=O, 3058 N–H str. of secondary amide (associated), 3213 N–H str. of imidazole; ^1^HNMR (DMSO-d_6_) δ: 3.33 (s, 2H of methylene), 6.91–7.70 (m, 8H aromatic), 6.89 (s, CH of thiazolidinone), 7.95 (s, NH of amide), ^13^C-NMR (DMSO-d_6_) δ: 35.74 CH_2_ of thiazolidinone, 39.76 CH_2_ aliphatic, 40.02 CH of thiazolidinone, (109.44, 116.50, 118.15, 119.56, 122.25, 130.36, 130.80, 133.19, 158.60) C aromatic, 162.26 C=O of thiazolidinone, 162.75 C of amide; ESI–MS (m/z) [M + 1]^+^ 456.53; Anal. Calcd. for C_22_H_25_N_5_O_2_S_2_: C, 58.00; H, 5.53; N, 15.37; O, 7.02; S, 14.08. Found: C, 57.97; H, 5.57; N, 15.39; O, 7.06; S, 14.03.

#### 2-(1H-Benzo[d]imidazol-2-ylthio)-*N*-(2-(4-(dimethylamino)phenyl)-4-oxothiazolidin-3-yl) acetamide (**5o**)

Yield 64.6%; mp 85–87 °C; R_f_ 0.60 (Toluene:Ethyl acetate: 3:1); IR (KBr cm^−1^) ν_max_: 746 OCN deformation of amide, 1362 C–N str. aryl tertiary amine, 1524 ring str. of thiazolidinone, 1600 C=O of thiazolidinone, 3050 N–H str. of secondary amide (associated), 2911 N–H str. of imidazole; ^1^HNMR (DMSO-d_6_) δ: 3.33 (s, 2H of methylene), 6.59 (s, CH of thiazolidinone), 6.65–7.96 (m, 8H aromatic), 8.49 (s, NH of amide); ^13^C-NMR (DMSO-d_6_) δ: 35.73 CH_2_ of thiazolidinone, 39.64 CH_2_ aliphatic, 40.13 CH of thiazolidinone, 40.84 CH_2_ of amide, (109.43, 111.63, 121.52, 124.99, 126.43, 128.22, 129.58, 151.18, 151.91, 153.25) C aromatic, 162.25 C=O of thiazolidinone, 168.41 C of amide; ESI–MS (m/z) [M + 1]^+^ 401.45; Anal. Calcd. for C_18_H_16_N_4_O_3_S_2_: C, 53.98; H, 4.03; N, 13.99; O, 11.99; S, 16.01. Found: C, 53.95; H, 4.07; N, 14.03; O, 11.96; S, 16.03.

#### 2-(1H-Benzo[d]imidazol-2-ylthio)-*N*-(2-(4-(diethylamino)phenyl)-4-oxothiazolidin-3-yl) acetamide (**5p**)

Yield 91.7%; mp 128–130 °C; R_f_ 0.38 (Toluene:Ethyl acetate: 3:1); IR (KBr cm^−1^) ν_max_: 744 OCN deformation of amide, 1357 C–N str. aryl tertiary amine, 1523 ring str. of thiazolidinone, 1633 C=O of thiazolidinone, 2970 N–H str. of imidazole; ^1^HNMR (DMSO-d_6_) δ: 3.33 (s, 2H of methylene), 6.95–7.91 (m, 8H aromatic), 6.58 (s, CH of thiazolidinone), 7.95 (s, NH of amide); ^13^C-NMR (DMSO-d_6_) δ: 12.39 C of CH_2_ CH_3_, 35.73 CH_2_ of thiazolidinone, 39.92 CH_2_ aliphatic, 43.53 CH of thiazolidinone, 39.92 CH_2_ of amide, 43.93 C of CH_2_CH_3_ (99.47, 110.93, 111.36, 120.72, 123.67, 127.73, 128.51, 129.87, 148.46, 153.42) C aromatic, 162.24 C=O of thiazolidinone, 189.39 C of amide; ESI–MS (m/z) [M + 1]^+^ 428.52; Anal. Calcd. for C_20_H_21_N_5_O_2_S_2_: C, 56.18; H, 4.95; N, 16.38; O, 7.48; S, 15.00. Found: C, 56.15; H, 4.97; N, 16.43; O, 7.46; S, 15.03.

#### 2-(1H-Benzo[d]imidazol-2-ylthio)-*N*-(4-oxo-2-styrylthiazolidin-3-yl)acetamide (**5q**)

Yield 83.2%; mp 210–212 °C; R_f_ 0.56 (Toluene:Ethyl acetate: 3:1); IR (KBr cm^−1^) ν_max_: 746 OCN deformation, amide present, 1493 ring str. of thiazolidinone, 1593 C=O of thiazolidinone, 3057 N–H str. of secondary amide (associated), 3206 N–H str. of imidazole; ^1^HNMR (DMSO-d_6_) δ: 3.33 (s, 2H of methylene), 6.58 (d, 2H of CH=CH aliphatic, J = 12 Hz), 6.92 (s, CH of thiazolidinone), 6.99–7.95 (m, 8H aromatic), 8.06 (s, NH of amide); ^13^C-NMR (DMSO-d_6_) δ: 35.74 CH_2_ of thiazolidinone, 39.77 CH_2_ aliphatic, 39.90 CH of thiazolidinone, (125.72, 126.80, 127.18, 128.56, 128.81) C aromatic, 162.26 C of amide; ESI–MS (m/z) [M + 1]^+^ 411.47; Anal. Calcd. for C_20_H_18_N_4_O_2_S_2_: C, 58.52; H, 4.42; N, 13.65; O, 7.79; S, 15.62. Found: C, 58.55; H, 4.47; N, 13.63; O, 7.76; S, 15.66.

#### 2-(1H-Benzo[d]imidazol-2-ylthio)-*N*-(2-(4-hydroxynaphthalen-1-yl)-4-oxothiazolidin-3-yl) acetamide (**5r**)

Yield 74.4%; mp 237–239 °C; R_f_ 0.82 (Toluene:Ethyl acetate: 3:1); IR (KBr cm^−1^) ν_max_: 746 OCN deformation of amide, 1464 ring str. of thiazolidinone, 1599 C=O of thiazolidinone, 3055 N–H str. of secondary amide, 3226 N–H str. of imidazole; ^1^HNMR (DMSO-d_6_) δ: 3.33 (s, 2H of methylene), 6.96–7.95 (m, 8H aromatic), 6.91 (s, CH of thiazolidinone), 8.03(s, NH of amide); ^13^C-NMR (DMSO-d_6_) δ: 35.74 CH_2_ of thiazolidinone, 39.92 CH_2_ aliphatic, 40.83 CH of thiazolidinone, 39.78 CH_2_ of amide, (109.44, 128.89) C aromatic, 163.61 C=O of thiazolidinone, 168.24 C of amide; ESI–MS (m/z) [M + 1]^+^ 451.51; Anal. Calcd. for C_22_H_18_N_4_O_3_S_2_: C, 58.65; H, 4.03; N, 12.44; O, 10.65; S, 14.23. Found: C, 58.69; H, 4.07; N, 12.42; O, 10.69; S, 14.26.

### Antimicrobial activity evaluation

#### Determination of MIC

The in vitro antimicrobial potential of the synthesized derivatives was assessed using tube dilution method. The micro-organisms used in the study are *E. coli* (Gram-negative bacterium); *B. subtilis* MTCC 2063, *S. aureus* MTCC 2901 (Gram-positive bacterial strain); *C. albicans* MTCC 227 and *A. niger* MTCC 8189 (fungal strains) [24]. Serial dilutions of both standard and test compounds were prepared in double strength nutrient broth I.P. (Indian Pharmacopoeia) for bacterial strain and Sabouraud dextrose broth I.P. for fungi [25]. The bacterial cultures were incubated at 37 ± 2 °C for 24 h. The incubation temperature and period for *C. albicans* was 37 ± 2 °C for 48 h while for *A. niger* was 25 ± 2 °C for 7 day. The results of antimicrobial activity were compared to the standard antibacterial (norfloxacin) and antifungal (fluconazole) drugs and are expressed in terms of MIC (minimum inhibitory concentration).

#### Determination of MBC/MFC

The subculturing of 100 µl of culture from each tube that showed no growth in MIC determination onto sterilized petri-plates containing fresh agar medium gave the minimum bactericidal concentration (MBC) and fungicidal concentration (MFC) of the synthesized compounds. After incubation under suitable conditions of temperature and time, the petri-plates were analyzed for microbial growth visually. MBC and MFC denote the minimum quantity of a drug needed to kill nearly 99.9% of the microbes [26].

#### In vitro cytotoxic evaluation

The cytotoxicity of the synthesized benzimidazole-substituted-1,3-thiazolidin-4-ones was evaluated in vitro on human colorectal carcinoma (HCT116) using Sulforhodamine-B (SRB) assay and the results were compared with that of the standard anticancer drug, 5-fluorouracil. This method is highly cost effective allowing testing of a large number of samples within a short period of time as compared to fluorometric methods [27]. The results of anticancer activity are expressed in terms of µM/ml.

The cells were allowed to attach to the walls of 96-multititre plates for a period of 24 h before treatment with the test compounds. Solutions of the test and standard compounds were prepared in DMSO and made up to appropriate volume with saline. Monolayer cells with different concentrations (5, 12.5, 25 and 50 µg/ml) of the test compounds were then incubated at 37 °C for 48 h in an atmosphere of 5% carbon dioxide. The cells were fixed with trichloroacetic acid for an hour, washed with water and stained with 0.4% w/v solution of pink colored aminoxanthine dye, Sulforhodamine-B, in acetic acid for 30 min. The cultures were washed with 1% acetic acid to remove the excess stain. The attached stain was recovered using Tris-EDTA buffer. The colour intensity was measured using ELISA reader. The experiment was done in triplicate.

#### Molecular docking studies on CDK-8

All the synthesized derivatives were docked onto the crystal structure of cyclin-dependent kinase 8 (CDK8) using sequential docking procedure on the crystal structure [PDB ID: 5FGK] retrieved from the protein data bank (PDB) [16]. The CDK8 protein structure was optimized using protein preparation wizard by removing the water molecules, hetero-atoms and co-factors. Hydrogen, missing atoms, bonds and charges were computed through Maestro. The synthesized benzimidazole-substituted-1,3-thiazolidin-4-ones were further docked. The structures of synthesized derivatives were built and optimized using LigPrep module implemented in Schrodinger Maestro. Ligand preparation includes generating various tautomers, assigning bond orders, ring conformations and stereochemistry. All the generated conformations were minimized using OPLS2005 force field prior to docking study.

A receptor grid was generated around the active site of CDK8 enzyme by choosing centroid of the enzyme complexed ligand (5XG ligand taken as the reference). The size of grid box was set to 20 Å radius using receptor grid generation implemented in Glide [28]. Docking calculations were accomplished using Glide. All docking calculations were performed using Extra Precision (XP) mode. The Glide docking score determined the best docked structure from the output. The interactions of these docked complexes were further analyzed and imaged using PyMOL [29].

## Abbreviations

HCT116: human colorectal cell line
MIC: minimum inhibitory concentration
MBC: minimum bactericidal concentration
MFC: minimum fungicidal concentration
CRC: colorectal cancer
CDK8: cyclin dependent kinase-8
IR: infra-red spectroscopy
^1^HNMR: proton nuclear magnetic resonance
^13^CNMR: carbon nuclear magnetic resonance
*S. aureus*: *Staphylococcus aureus*
*B. subtilis*: *Bacillus subtilis*
*E. coli*: *Escherichia coli*
*C. albicans*: *Candida albicans*
*A. niger*: *Aspergillus niger*
